# Intraoperative indocyanine green fluorescence guidance for excision of nonpalpable breast cancer

**DOI:** 10.1186/s12957-016-1014-2

**Published:** 2016-10-18

**Authors:** Jintao Liu, Wenbin Guo, Meng Tong

**Affiliations:** Department of Surgery, The Breast Center, Dalian Central Hospital, Dalian Medical University, No. 826, Xinan Road, Shahekou District, Dalian, 116033 China

**Keywords:** Nonpalpable breast cancer, Indocyanine green fluorescence imaging, Intraoperative excision

## Abstract

**Background:**

Different techniques have been used for the guidance of nonpalpable breast cancer (NBC), but none of them has yet achieved perfect results. The aim of this study was to evaluate the feasibility of indocyanine green (ICG) fluorescence-guided nonpalpable breast cancer lesion excision (IFNLE), to introduce an alternative technique.

**Methods:**

The data about 56 patients with preoperatively diagnosed NBCs operated with the help of intraoperative IFNLE between November of 2010 and September of 2014 were retrospectively analyzed.

**Results:**

ICG fluorescence localized all lesions at surgery. Re-excision due to positive resection margins was necessary in two patients (3.6 %; 2/56) with ductal carcinoma in situ (DCIS) at the surgical margins. Mastectomy was necessary in one patient (1.8 %; 1/56) due to multifocal invasive carcinoma. The mean volume of the excised tissue was 38.2 ± 16.5 cm^3^.

**Conclusions:**

IFNLE is a technically applicable and clinically acceptable procedure whenever a breast cancer needs image-guided excision.

## Background

Breast cancer diagnosis has undergone a dramatic evolution in the last few decades. Due to the emergent availability in mammography screening programs, there has been a rapid increase in the diagnosis of nonpalpable breast cancer (NBC) to the breast surgeon [1]. Diagnosis is therefore more often made at an early stage, which is associated with decreased incidence of lymph node involvement. A suitable treatment for early stage breast cancer is breast-conserving surgery (BCS) [2, 3].

For breast-conserving treatment of NBC, an accurate intraoperative guidance is essential to achieve complete tumor removal with acceptable cosmetic results [4]. Traditionally, the surgical excision of this kind of lesion has been carried out with some methods; however, each technique suffers from various limitations [5–7]. Recently, a novel method of using indocyanine green (ICG) fluorescence has been described in breast surgery [8, 9].

In this article, we designed our study to evaluate the usefulness of ICG fluorescence-guided nonpalpable breast cancer lesion excision (IFNLE) and its results, to see whether the technique can be accepted as an alternative method of image guidance for the procedure.

## Methods

### Sample collection

Between November of 2010 and September of 2014, 56 patients with histologically diagnosed NBCs who underwent IFNLE were included in this retrospective analysis. Patients of benign lesions removed with the help of preoperative ultrasonography (US) were excluded from the study. Written consent was obtained from all participants, and the study was approved by the institutional ethics committee of Dalian Central Hospital before the operation.

### Lesion localization

**Fig. 1:**
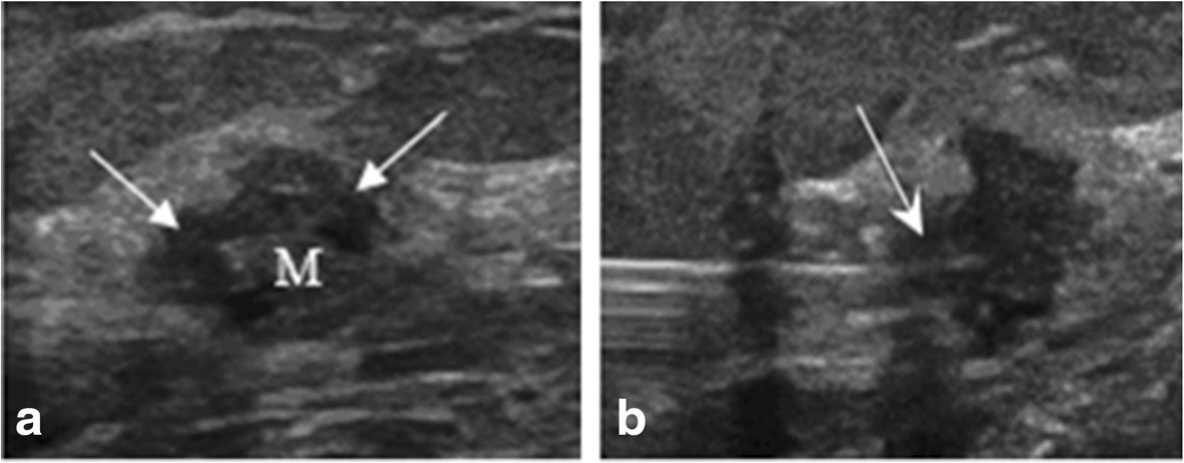
Ultrasonographic appearance of a nonpalpable breast lesion: **a** hypoechoic with irregular margins; **b** intralesional injection of indocyanine green (ICG) solution with a spinal needle under ultrasound guidance (*arrow*)

Before surgery, image guidance was done by US with a 10-MHz linear transducer (Voluson 730 Expert; GE Healthcare, Chalfont St Giles, UK). Breast US provided specific information regarding the tumor location, extent and its longest diameter, the tumor depth from the skin, and distance from the pectoralis muscle. Under local anesthesia, a 22-gauge spinal needle was placed through the lesion long axis into its center, and 2 ml (10 mg) of ICG solution (Diagnogreen, Daiichi pharmaceutical, Tokyo, Japan) was injected intralesionally (Fig. 1). Correct localization was confirmed by US with the help of the dynamic change in echogenicity of the lesion after ICG injection.

### ICG fluorescence-guided breast lesion excision

**Fig. 2:**
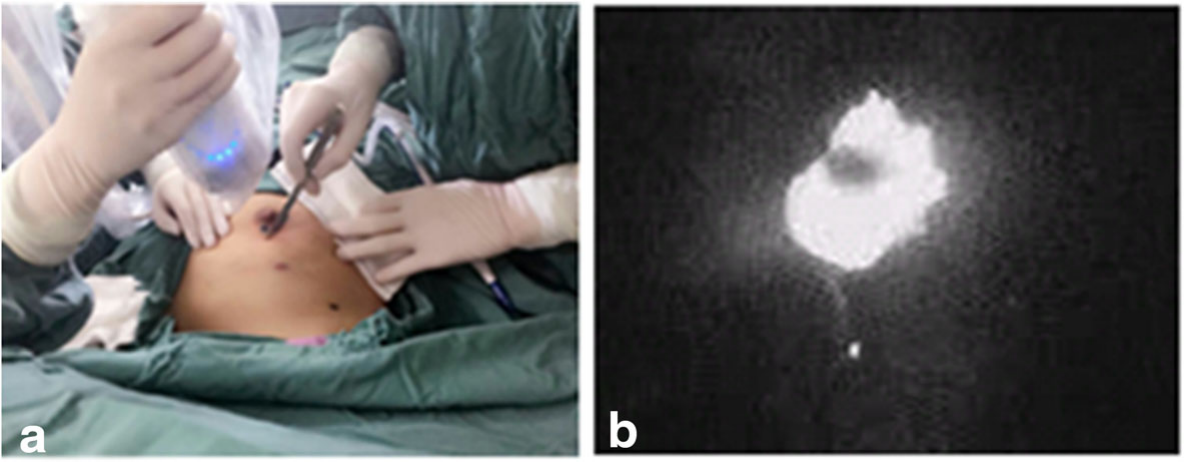
**a** During surgery, a near-infrared-sensitive imaging system was used to observe. **b** The area of ICG-derived fluorescence

**Fig. 3:**
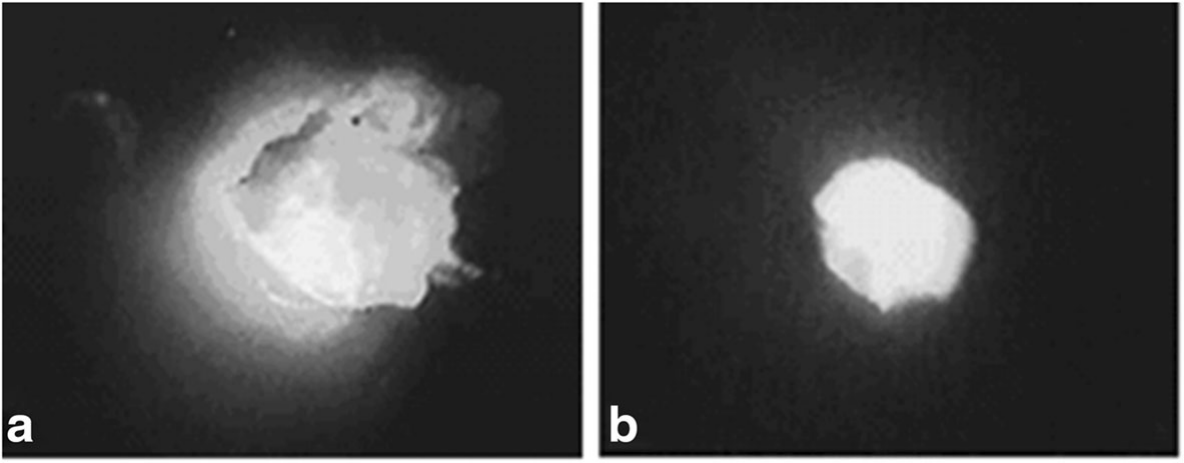
**a** The resection margins were defined under the guidance of fluorescence imaging; **b** appearance of the specimen after excision

The photodynamic eye (PDE; manufactured by Hamamatsu Japan) was used as the intraoperative ICG fluorescence-guided device. After the surgical lights were turned off, the surgeon localized the tumor under PDE guidance and determined in an incision line. The surgeon identified and marked the site of the skin incision with a suitable pen by observing the area of ICG fluorescence. During the surgery, the first assistant held the PDE at a distance of 20–30 cm from the breast (Fig. 2). Following the skin incision, dissection was carried out toward the injected ICG covering the lesion, and the resection margins were defined with fluorescence imaging (Fig. 3). After the lesion was excised, the PDE was used to examine the bed to verify that there were no residual areas of fluorescence. If there was any site that emits a strong fluorescence, that site was removed along with the surrounding tissue. If the lesion depth from the skin was more than 10 mm, a second injection with a smaller dose of ICG solution (0.2 ml) was placed into the subcutaneous tissue projection of the lesion in order to achieve a better visualization of the fluorescence during the surgery.

All surgical specimens underwent mammographic or US study to verify a complete removal of lesions within the specimen. The all excised samples were also marked with blue and black ink to orientate the anterior and posterior surfaces, respectively. Histological examination of the specimens was performed at the pathology department. Thorough examination included the measurement of the specimen in three dimensions, evaluation of the greatest tumor diameter, and margin status with documentation of the closest margin in addition to the determination of the final histological diagnosis. All resected tissue was evaluated by standard hematoxylin and eosin staining.

## Results

US localization took very little time (8–10 min). The result of preoperative diagnostic US for tumor dimensions was satisfactory. The sizes of the lesions measured on the US ranged from 0.6 to 2.1 cm (mean ± SD, 1.3 ± 0.5 cm). Patients and tumor characteristics are listed in Table 1. The ICG-guided localization correctly identified all target lesions at the initial surgery (there was no missed lesion), yielding a 100 % positive predictive value for the lesions. There was no false-positive localization.

**Table 1:** Patient and tumor characteristics

Characteristic	Value and percentage
No. of patients	56
Median age (range), years	53.8 (34–78)
Tumor diameter, mean ± SD (cm)	0.6–2.1 cm, 1.3 ± 0.5
Grade	
1	10 (17.8 %)
2	31 (55.4 %)
3	15 (26.8 %)
Accurate location of breast cancer	56 (100 %)
Margins of excision	
Satisfactory	53 (94.6 %)
Unsatisfactory	3 (5.4 %)
Tumor type on US examination	
DCIS	2 (3.6 %)
Invasive carcinoma	54 (96.4 %)
Tumor type on pathological examination	
DCIS	6 (10.7 %)
Invasive carcinoma	50 (89.3 %)
Node status (both physical and US examination)	
Negative nodes	45 (80.4 %)
Positive nodes	11 (19.6 %)

*DICS* ductal carcinoma in situ

Satisfactory margins (defined as no re-excision necessary) were found in 94.6 % (53/56) of patients. Re-excision due to positive resection margins was necessary in two patients with ductal carcinoma in situ (DCIS) at the surgical margins. In addition, mastectomy was necessary in one patient 1.8 % (1/56) due to multifocal invasive carcinoma unknown at time of ICG-guided excision. Thus, two patients required surgical re-excision, one required mastectomy, and the re-excision rate for all ICG-guided procedures was 5.4 % (3/56). There was an additional patient who had focal carcinoma at a margin and did not have further surgery, and she was an elder woman who had five clear margins of her 15-mm invasive carcinoma and refused re-excision for an associated focus of in situ carcinoma at one margin.

The volume of the excised tissue was calculated from the three dimensions measured by the pathologist using the formula 4/(3π) × a × b × c. The mean volume of the excised tissue was 38.2 ± 16.5 cm^3^ (Table 2).

**Table 2:** Marginal status of excision and volumes of the specimens in the patients

Characteristic	Histological diagnosis in re-excision specimen	*n* (%)	Mean volume ± SD (cm^3^)
No re-excision (including one focal positive case)		53 (94.6 %)	
Re-excision		3 (5.4 %)	
	DICS	2 (3.6 %)	
	Multifocal invasive cancer (mastectomy)	1 (1.8 %)	
Excision specimens			38.2 ± 16.5

*DICS* ductal carcinoma in situ

On pathological examination, there were six patients (10.7 %; 6/56) with DCIS and the remaining 50 patients (89.3 %; 50/56) had invasive disease. US identified only two patients with DCIS (33.3 %; 2/6). Thus, DCIS could not be visible by US in four patients (66.7 %; 4/6).

The mean follow-up was 19 months (range, 6–38 months). Patients underwent physical examination and mammogram of the affected breast every 3 months for the first 2 years. Thereafter, the same examinations occurred every 6 months. During the time of follow-up, no patient had local or systemic recurrence.

## Discussion

To date, several different techniques have been described for the localization of nonpalpable breast lesion [10–12], the most common being the introduction of a wire-guided localization (WGL) [6]. The downsides of this method are the displacement or deviation of the wire, the possibility of re-intervention due to positive margin, and patient discomfort [6, 13]. Alternatively, radio-guided occult lesion localization (ROLL) is usually performed by a radiologist immediately before the surgical procedure [5]. Zgajnar et al. [14] reported smaller excisions with a higher percentage of clear surgical margins in ROLL compared with WGL. However, even with ROLL, they still achieved clear margins in 70 % of 56 breast cancer patients. Otherwise, limitations of this process are many. Scheduling patients both in radiology and then in surgery may be difficult, and the technique is impractical at institutions where there is no nuclear medicine facility [5].

It is, therefore, not surprising that alternative methods for tumor guidance excision, such as US, have been investigated [7]. In a comparative study of three different methods used in BCS of nonpalpable lesions in 201 patients, Krekel et al. [15] found a significantly lower rate of positive margins with the use of US guidance (3.7 %) compared with wire localization (21.5 %) and ROLL (25 %). Similarly, re-excision rates of from 3.2 to 11.4 % after intraoperative US guidance of nonpalpable lesions have been reported by other authors (Table 3) [15–20].

**Table 3:** Intraoprative ultrasound and wire-guided localization for nonpalpable breast lesions

Author	Design	Patients *n*		Re-excision *n* (%)		Mean excision volume (cm^3^)	
		US	WGL	US	WGL	US	WG
Krekel [15]	Retrospective	52	117	3.7	21.3	71.1	54.9
Sikošek [16]	Retrospective	125	N.R.	3.2	N.R.	42.1	N.R.
Sheikh [17]	Prospective	57	N.R.	8.7	N.R.	N.R.	N.R.
Barentsz [18]	Prospective	138	120	6.7	6.5	62.8	56.6
James [19]	Retrospective	96	39	11.4	11.9	N.R.	N.R.
Bennet [20]	Prospective	115	43	7	17	56.5	63.9

*US* intraoperative ultrasound, *WGL* wire-guided localization, *N.R.* not reported

ICG fluorescence has been used as a tracer for many years and has demonstrated excellent safety profile for clinical use [21, 22]. The principle of fluorescence measurement is as follows: Injected ICG molecules are promptly bound to the globulin fractions in serum proteins. Under this condition, when excitation light of 760 nm is irradiated, fluorescence with a peak wavelength of 845 nm will be emitted. This process is captured by a fluorescence navigation system using a PDE. Fluorescence at a depth of 10–15 mm beneath the skin can be visualized, based on the distance that the light is able to reach [23].

Today, most of the reports for ICG fluorescence applied to breast cancer are about the intraoperative identification of sentinel lymph node (SLN). The technique is feasible and accurate with acceptable sensitivity and specificity, comparable to conventional node detection methods [23, 24]. Meanwhile, there are theoretical and practical advantages about the fluorescence navigation technique as a guidance to the surgical excision of NBCs. However, the only report about IFNLE was described in 2012 by Aydogan F and associates [25], who used the technique as a guidance for the excision of NBCs in two cases, and had achieved clear margins. This study summarizes our experience using the method in 56 cases.

The presented method, which we termed IFNLE, shares the similar technical principle with ICG-guided SLN biopsy. The key point of IFNLE is that ICG is injected directly into the center of the lesion under US localization. Subsequently, dissection is carried out, and resection margins are defined by observing the fluorescence areas with a near-infrared camera. One could theorize that real-time imaging during the whole procedure allows for a very accurate determination of margins around the tumor by direct visualization of ICG fluorescence. By using this technique, the breast lesions were correctly localized, the fluorescent areas corresponded well to the sites of lesion, and we excised all of the breast lesion successfully just one time (Fig. 3). If the breast lesion was located deeper, for example more than 10 mm, in addition to intralesional injection, a much smaller dose of ICG (0.2 ml) can also be injected into the subcutaneous tissue projection of the lesion in order to enhance fluorescence visualization.

In our study, re-excisions were required for positive margins in only two patients (3.6 %) and mastectomy was necessary in one patient (1.8 %) (Table 2). Thus, the re-excision rate for all IFNLE procedures was 5.4 % (3/56) which is comparable to other series of US localization and better than that of WGL (Table 3). The pathological finding in three patients required re-excisions with one calcified DCIS, one noncalcified DCIS at the surgical margins, and multifocal invasive carcinoma in the one case (underwent mastectomy). This conforms to Cary et al. [11] who found that regardless of the type of imaging guidance, patients with DCIS or multifocal invasive cancer will be at increased risks for a positive margin, re-excision, or mastectomy.

Until now, the volumes of excised specimens were reported in only a few studies with nonpalpable lesions. The mean total resection volume ranged from 42 to 71 cm^3^ with US and 54 to 63 cm^3^ with WGL (Table 3). Because US guidance and excision of the lesion cannot be performed simultaneously and there is no guide wire to see or feel during the procedure, it may be also difficult for a surgeon to define the tumor margins accurately, especially for women with large breasts and deeply seated lesions. In order to ensure a negative margin, the surgeon usually excised a wide and deep margin around the tumor, leading to excessive tissue excision [26, 27]. Owing to the displacement of the wire or inaccurate wire localization, unnecessary large volumes of tissue will be removed by WGL technique [6, 13].

Otherwise, the PDE need not touch the breast by the ICG fluorescent guidance; it can constantly provide a direct visualization during the whole excision, which has the potential to overcome the weak points of US or WGL [25]. In the present study, the mean resection volume is only 38 cm^3^ which indicates that a small amount of the healthy breast tissue unnecessarily sacrificed was removed. We have demonstrated that IFNLE method enables the resection of smaller volumes of the tissue with a low re-excision rate. So another main benefit of the technique is the ability to limit the amount of excised normal tissue while achieving adequate distance from the lesion to the surgical margin, and therefore, specimen volume will be minimized. This is important not only for the success rate of the procedure but also for the cosmetic outcome in breast-conservation treatment [28, 29].

The benefits of IFNLE are as follows: (1) the site of the skin incision can be defined precisely; (2) infrared fluorescence image makes it easy to recognize the resection margins of the breast lesion; and (3) the method of IFNLE facilitates visualization of the whole process of excising the lesion in real time during surgery, and the volume of resection may be smaller.

However, the method also has several problems such as (1) this method cannot be applied to patients with hypersensitivity to iodine because the commercially available product contains approximately 5 % sodium iodides as a contaminant and (2) ICG tattooing lasted 10 to 14 days before disappearing spontaneously [8].

Our study also has several limitations. Most importantly, it is a retrospective series of observation without a control group. Though some advantages of IFNLE seem obvious, a prospective randomized study is needed to gain more reliable information, and we are planning to conduct such a study in the future.

## Conclusions

IFNLE proved to be a reliable and very useful tool to excising NBCs, improving the process of image-guided surgery. It is accurate and technically feasible, and the re-excision rate is lower with less the volume of healthy breast tissue unnecessarily sacrificed. It simplifies organizational work and spares the patient from discomfort of preoperative needle localization and drawbacks of US-guided excision or ROLL. Although further studies assessing the feasibility and validation of this technique are required, intraoperative ICG-guidance should be considered whenever a breast cancer needs image-guided excision.

## Abbreviations

BCS: Breast-conserving surgery
DCIS: Ductal carcinoma in situ
ICG: Indocyanine green
IFNLE: ICG fluorescence-guided nonpalpable breast cancer lesion excision
NBC: Nonpalpable breast cancer
OLL: Radio-guided occult lesion localization
PDE: Photodynamic eye
SLN: Sentinel lymph node
US: Ultrasonography
WGL: Wire-guided localization
